# Considerations for the Implementation of Massively Parallel Sequencing into Routine Kinship Analysis

**DOI:** 10.3390/genes16030238

**Published:** 2025-02-20

**Authors:** Lucinda Davenport, Laurence Devesse, Somruetai Satmun, Denise Syndercombe Court, David Ballard

**Affiliations:** King’s Forensics, Department of Analytical, Environmental and Forensic Sciences, Faculty of Life Sciences and Medicine, King’s College London, London SE1 9NH, UK; laurence.a.devesse@kcl.ac.uk (L.D.); somruetai.satmun@kcl.ac.uk (S.S.); denise.syndercombe-court@kcl.ac.uk (D.S.C.); david.ballard@kcl.ac.uk (D.B.)

**Keywords:** kinship analysis, massively parallel sequencing, next generation sequencing, kinship casework, sequence-based STR analysis, microhaplotypes, population databases, sequence allele nomenclature, likelihood ratio, forensic genetics

## Abstract

*Background*: Investigating the way in which individuals are genetically related has been a long-standing application of forensic DNA typing. Whilst capillary electrophoresis (CE)-based STR analysis is likely to provide sufficient data to resolve regularly encountered paternity cases, its power to adequately resolve more distant or complex relationships can be limited. Massively parallel sequencing (MPS) has become a popular alternative method to CE for analysing genetic markers for forensic applications, including kinship analysis. Data workflows used in kinship testing are well-characterised for CE-based methodologies but are much less established for MPS. When incorporating this technology into routine relationship casework, modifications to existing procedures will be required to ensure that the full power of MPS can be utilised whilst maintaining the authenticity of results. *Methods*: Empirical data generated with MPS for forensically relevant STRs and SNPs and real-world case experience have been used to determine the necessary workflow adaptations. *Results*: The four considerations highlighted in this work revolve around the distinctive properties of sequence-based data and the need to adapt CE-based data analysis workflows to ensure compatibility with existing kinship software. These considerations can be summarised as the need for a suitable sequence-based allele nomenclature; methods to account for mutational events; appropriate population databases; and procedures for dealing with rare allele frequencies. Additionally, a practical outline of the statistical adjustments required to account for genetic linkage between loci, within the expanded marker sets associated with MPS, has been presented. *Conclusions*: This article provides a framework for laboratories wishing to implement MPS into routine kinship analysis, with guidance on aspects of the data analysis and statistical interpretation processes.

## 1. Introduction

Kinship analysis is an important application of forensic DNA typing, and often employs similar methodologies to those used in criminal investigations, such as short tandem repeat (STR) typing with capillary electrophoresis (CE).

Kinship investigations examine the proportion of genetic information shared between individuals in order to infer a genetic relationship between them. The International Society of Forensic Genetics (ISFG) recommends that a likelihood ratio (LR) should be used to evaluate the most likely pedigree in a kinship scenario [1]. To understand the likelihood of a given pedigree, an alternative pedigree must be considered to give context to the calculation. For example, when trying to determine if two individuals are related as half-siblings, it is useful to consider how much more likely genetic data (i.e., STR genotypes) are if they were related as half-siblings than if they were unrelated. In order to calculate the probability of observing these genotypes in two unrelated people (i.e., any alleles in common are shared purely by chance), it is necessary to determine how common an allele is within a population group of interest. Therefore, during a kinship investigation, certain data is required: observed genotypes of tested individuals (genetic data), pedigree hypotheses, and population data in the form of allele frequencies. Additionally, other relevant parameters may be needed, such as the recombination rates between linked loci or mutation rates in cases where marker exclusions have been observed.

In its simplest form, such as for a paternity case, a likelihood ratio (or paternity index) calculation can be carried out by hand [2]. Whilst in theory these formulae can be expanded to deal with more distant relationships, the computational time, complexity, and chance of human error will increase greatly. Therefore, dedicated software solutions that employ computerised algorithms have been developed to cope with calculations involving increased pedigree sizes or complexity and multiple alternative pedigrees.

There are a number of available software options that have varying levels of sophistication and capability, including modules already integrated into laboratory management systems (LIMS). Two such programs commonly used in kinship testing laboratories [3] that will be discussed in this work are Familias [4] and FamLink2 [5,6,7]. Both are freely available to download from https://familias.no/ and https://famlink.se/f_index.html, respectively. Familias employs an algorithm that can successfully calculate likelihoods for large pedigrees and large numbers of unlinked genetic markers. One of the main drawbacks of Familias is that the software is unable to account for genetic linkage between loci. As linked markers have a reduced chance of recombination occurring between them, this co-segregation of closely located loci means that they can no longer be treated as independent in certain calculations. FamLink was developed as a user-friendly software that is capable of dealing with linkage corrections for likelihood calculations. FamLink has been designed to complement Familias, meaning that the programs can be used in tandem when analysing case data.

One main limitation of CE-based DNA typing methods when applied to kinship testing is the restricted marker multiplexing capacity for analysing multiple STR loci at once. At present, CE STR multiplex kits, on average, target up to 23 autosomal STR loci. The need for size- and dye-based separation of all identified alleles limits the number of loci that can be targeted simultaneously. This means that although CE-based STR kits often provide enough information to resolve paternity cases, they are much less likely to be able to determine more distant or complex pedigrees, such as those involving second- or third-degree relationships, especially when using a single assay alone [8,9,10]. Therefore, there is a need for expanded marker sets in order to resolve these more complex or distant relationships with more statistical confidence, i.e., to obtain larger LR values.

Whilst a common approach to improve relationship resolution with CE is to carry out analysis with additional multiplex systems to obtain data from a larger number of STR loci, currently, it is only possible to target up to ~50 autosomal STR loci when combining data from multiple commercially available kits. The resolving power of this number of STRs can still prove insufficient for many cases.

Massively parallel sequencing (MPS) has been presented as an alternative method for targeting forensically relevant genetic markers, such as STRs, as well as single nucleotide polymorphisms (SNPs) [11]. MPS does not rely on the separation of alleles based on size; therefore, many more markers can be multiplexed together, meaning that more information can be obtained from a single run in comparison to CE-based STR or SNP analyses [12,13]. MPS can also provide advantages over CE-based approaches in kinship scenarios involving poor quality DNA samples, such as investigations related to the indirect identification of human remains using familial references. In addition to the ability to target smaller SNP amplicons, the size of many STR amplicons can also be reduced in comparison to their CE counterparts; these characteristics make MPS useful for the analysis of degraded DNA [14,15,16].

A further benefit of MPS is the generation of sequence-level data. This means that a more in-depth interrogation of genetic loci is possible in comparison to electrophoretic methods. The presence of sequence variation between STR alleles of the same length and additional point variants within SNP amplicons greatly increases distinguishable allelic diversity and the overall power of discrimination of the marker set [17,18,19]. The increased number of alleles that can be distinguished in turn reduces the frequency at which each allele will appear in a population. This can be beneficial for kinship analysis, as the presence of such an allele in two individuals’ DNA profiles will have the potential to provide high evidential weight when determining if they are related [8]. Furthermore, it decreases the likelihood of unrelated individuals sharing alleles by chance due to the commonness of the allele in a population.

However, to utilise the information gained from this variation, appropriate population studies need to be carried out in order to estimate the frequency of all observed alleles. The ForenSeq DNA Signature Prep kit (Verogen-Qiagen) [20] is an example of an MPS assay targeting both STR and SNP markers that has been well-characterised within the forensic community [17,18,19,21,22,23,24,25,26,27,28,29,30,31,32].

Before new analytical processes can be implemented into routine forensic casework, laboratories are required to validate the methods against relevant standards, such as ISO:17025. Despite the novelty of MPS analysis within the forensic field, the Scientific Working Group on DNA Analysis Methods (SWGDAM) has also published additional interpretation guidelines for STR typing with MPS [33] and, more recently, typing of SNPs with MPS [34]. These guidelines set out the key criteria that laboratories must address in their internal validations, including but not limited to establishing analytical, interpretation, and stochastic thresholds, determining robust locus and allele designators, and formulating methods to evaluate sequencing run quality.

This article aims to highlight the specific additional factors that need to be considered when implementing MPS technology into a kinship analysis workflow. Sequence data generated from five UK-relevant population groups using the DNA Signature Prep kit (see publications [17,18,19] for details) and real-world casework experience has been used to demonstrate four considerations related to the data analysis aspects of kinship investigations. These considerations encompass the elements of kinship data workflows that differ from CE-based analysis, such as allele nomenclature, data compatibility with existing kinship software solutions, and the overall approach to likelihood ratio calculations. Practical solutions to address these issues have also been outlined.

## 2. Considerations

### 2.1. Sequence-Based Allele Genotyping and Nomenclature

**Consideration 1: When using sequence-level data, there is a need for a robust nomenclature system that allows for easy association of case genotypes with allele frequency databases whilst also being compatible with kinship software solutions.**

Genotyping samples from data produced using capillary electrophoretic methods has been extensively studied, with various commercial software solutions available. A popular example is ThermoFisher’s GeneMapper™ ID-X, which is a genotyping software specifically designed for use in forensics. With the aid of manufacturer-provided packages containing specified analysis parameters, including allele bins and user-definable interpretation thresholds, genotyping of reference level samples can be easily streamlined. This has allowed many casework laboratories to validate the method so that after processing sample genotype type data can be easily exported in a chosen format.

With the understanding that multiple laboratories will be using a similar procedure, specialist kinship software, such as Familias, has been designed so that the genotype file exported from GeneMapper can be directly imported into a Familias project. From there, other relevant data, including the tested pedigrees, can be defined within the project, a likelihood ratio can be calculated, and the results easily exported in various formats. Whilst each laboratory will need to validate their exact workflow using empirical data, a supportive scaffold for this process exists.

MPS is still a relatively new technology in the forensic field. Companies, such as Verogen and ThermoFisher, have tried to cater to laboratory needs in the form of integrated workflows, making it easier for customers to validate the entire process. For example, when analysing samples with the ForenSeq DNA Signature Prep kit via the Forensic Genomics application on the MiSeq FGx system, the sequence data is immediately uploaded to and analysed by the Universal Analysis Software (UAS). UAS acts in a similar manner to GeneMapper ID-X, providing an easy-to-use interface where allele calling thresholds are automatically employed, analysts can manually check the genotypes if required, and genotype data can be directly exported. This can be particularly useful as it removes the need for the analysts to have any bioinformatic experience. Up until recently, a major downside of this system has been that only length-based allele names can be exported. This meant that whilst full sequence information corresponding to each allele could also be exported, in order to convert this into a usable allele name, secondary processing was necessary. Currently, efforts by commercial providers are being made to incorporate sequence-based nomenclature into genotyping software solutions.

#### 2.1.1. STRs

**Figure 1:**
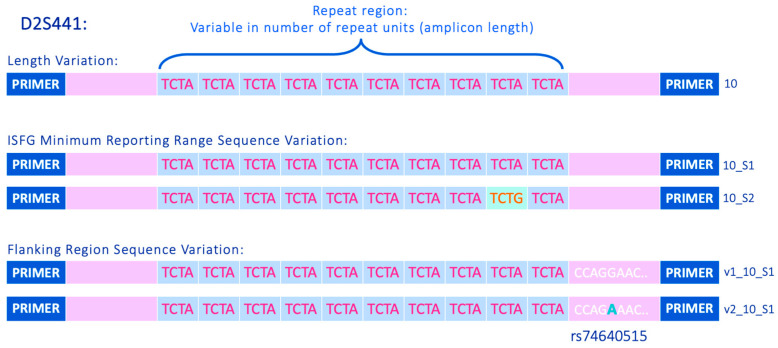
Example of sequence variation detected at STR locus D2S441. Allele “10” shows both repeat-region variation, shown by the colour change of the “TCTG” repeat unit, and flanking region variation, shown by an SNP change highlighted in turquoise. Alleles named using an internal naming system discussed in Section 2.1.3, where “SX” denotes the specific version of the sequence found in the ISFG minimum reporting range and “vX” denotes the version of the flanking region sequence.

When analysing STR loci with MPS, sequence variation has been shown to occur both in the previously established repeat-regions of the 27 autosomal STRs targeted by the ForenSeq DNA Signature Prep kit [17,21,22,23,24,25,26,27] as well as in the so called “flanking regions” that lie between the repeat-region of interest and the primer sequences [18,28,29,30,31]. Figure 1 gives an example of repeat-region and flanking region variation observed at the STR locus D2S441, a core marker used in forensic DNA typing. This highlights one of the key areas of debate surrounding the incorporation of sequence-based analysis of STRs into routine forensic casework—the need for a nomenclature system that can be used to describe these new sequence variants. An important requirement of the nomenclature system is back-compatibility with existing DNA databases (i.e., the length-based equivalent must be identifiable). Additionally, if including flanking region variation in the allele differentiation, appropriate allele ranges need to be defined with “start” and “stop” points, as well as the consistency of the directionality of a reported sequence (e.g., all loci reported in the forward orientation). With the increasing popularity of MPS in the forensic community, a number of naming systems, tools, and allele catalogues have been presented [35,36,37,38,39]. Guidelines and recommendations for STR sequence-based nomenclature going forward have been formulated and published by the DNA commission of the ISFG [40,41]. To aid data compatibility and reporting consistency between different MPS-based STR assays, the DNA Commission has defined a set of minimum reporting ranges for forensically relevant STR loci [41]. Furthermore, it has been proposed that condensing the corresponding minimum range sequences into a bracketed format using the STRNaming tool [35] is the most appropriate way to convert the reported sequences into a “human-readable” configuration. This algorithmic approach can also be applied to full amplicon sequences in order to maximise the informativeness of the genetic data obtained. This is of particular benefit for kinship applications. The STRNaming algorithm can be implemented online at https://www.fdstools.nl/strnaming/index.html; alternatively, it can be used via an offline version of FDSTools, however. This approach requires some level of bioinformatic skill.

Whilst bracketed sequences may be visually intuitive, there may be instances where a more concise short-hand allele name may be required. For example, some data analysis software solutions may not allow for special characters, such as brackets, or they may limit the total number of characters allowed within an allele name. As many of these analysis tools were originally developed to accommodate simple numeric names corresponding to STR allele length data generated by CE, they may be less able to recognise more complex multi-character names.

One such minimal “code”-based naming system that has been proposed within the community involves converting the DNA string sequence to a “Sequence Identifier” (SID) consisting of letters using a secure hashing algorithm [36]. Due to the way this hash function works, the SID code will be dependent on a pre-determined sequence range. Consequently, the SID generated from the minimum range sequence will bear no resemblance to the SID generated from the full amplicon sequence despite having the same core structure. As hashing is a unidirectional process, whilst each time the algorithm encounters the same sequence it will produce the same SID, there is no simple way of converting a SID into a sequence without the use of a user-curated sequence SID catalogue.

The chosen naming system for kinship applications must be functional and fit for purpose when used in population databases so that case genotypes can be easily linked to the associated allele frequencies. The allele designations must also be compatible with any kinship software solutions employed. To maximise the amount of genetic information that can be obtained from the sequence data whilst still allowing for comparison of data across MPS kits, the allele names should enable easy identification of both variation seen in the minimum sequence range as well as the full amplicon sequence. If making use of data outside of the minimum range, there needs to be consistency in the implemented data processing workflow to ensure that all samples in any given kinship investigation are typed uniformly. Furthermore, any naming system should ideally be simple enough to be used throughout the workflow without being unwieldy, thus allowing for effective data manipulation and reliable results.

#### 2.1.2. SNPs

MPS technology also allows for the enhanced analysis of SNP loci. Whilst traditionally SNP typing techniques only aimed to provide information about a single nucleotide position, MPS enables the collection of sequence information for an entire amplicon. Consequently, any variation in the flanking regions surrounding the SNP of interest can also be detected. A small number of studies have presented population data for the 94 SNP loci targeted by the ForenSeq DNA Signature Prep kit in the form of bi-allelic SNP allele frequencies only [26,27,28] or considering the genetic variation within the whole sequenced SNP amplicon [19,29,30,31,32].

The Universal Analysis Software (UAS) is the default data processing software implemented within a ForenSeq DNA Signature Prep workflow. More recent iterations of this software include the functionality to report full sequence data for the SNP amplicon directly via a “*Flanking Region Report*” rather than there being a requirement to re-process the raw data with external tools [29,30,31,32]. This provides easier access to the full amplicon sequences for users without a background in bioinformatics.

When two or more SNPs located within the same amplicon give rise to three or more distinct haplotypes, this can be referred to as a “microhaplotype”. Due to their multi-allelic nature, on a per-locus basis, microhaplotypes are likely to be more informative than bi-allelic SNPs, resulting in an increased interest in these loci within the forensic community. Recently, a microhaplotype working group (MWG) has been established with a particular aim of standardising the nomenclature of both microhaplotype loci and corresponding alleles, which is currently very varied within the published literature.

**Figure 2:**
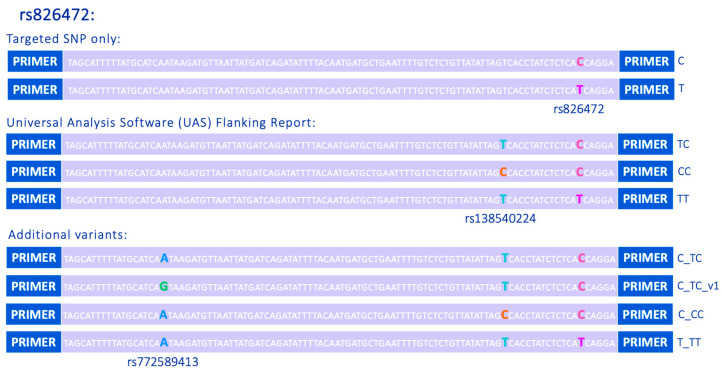
Example of sequence variation detected at SNP locus rs826472. Alleles “C” and “T” show variation at the chosen SNP of interest. Alleles “TC”, “CC”, and “TT” show variation at the selected sites reported in the UAS “Detected Bases”. Alleles determined by full amplicon sequence variation named using internal naming system where the bi-allelic allele name is followed by the UAS “Detected Bases” and “vX” denotes the version of the full amplicon sequence in relation to the UAS “Detected Bases” output.

The potential for microhaplotypes to be found within the DNA Signature Prep SNP loci was unlikely to be factored into the original marker selection. Their discovery is purely a beneficial consequence of MPS analysis and the ability to interrogate the sequence data for the entire amplicon (see Figure 2 for an example).

The more recent versions of UAS have been updated to report haplotypic allele names referred to as “Detected Bases” in the *Flanking Region Report*, where the nucleotide letters correspond to the combination of bases seen in the amplicon. The number and selection of bases included in the “Detected Bases” output were chosen by Verogen to represent the sites that are expected to show observable population variation; therefore, this number varies between loci, with seven being the maximum number of sites reported at any one locus (i.e., rs8078417, where the total amplicon length is 102 bp). Published work has also shown that considerable variation outside of these reported positions does exist [19]. As such, this additional variation is available yet rarely exploited; to make use of it, there is again a requirement to differentiate alleles and name them accordingly.

#### 2.1.3. Example Allele Naming System

From a kinship testing perspective, the allele naming system for both STRs and microhaplotypic SNP-based loci needs to be consistent within the implemented DNA workflow, assuming all samples will be processed by the same laboratory.

In addition to being compatible with any chosen kinship software solutions, laboratories will need relevant population data in the form of allele frequencies that correspond to the naming system employed in the genotyping workflow. It will be particularly beneficial for laboratories to make use of a system that can be easily adapted depending on the analysis method used, e.g., whether the full amplicon, STR minimum range, or single base of interest is analysed. A nested allele naming system where the designators reference these different analysis options is an ideal solution in this case. This will also be advantageous for cross-laboratory processing of casework, collaborative studies, and international proficiency tests designed to evaluate the interpretation of data generated with MPS.

During the process of incorporating the DNA Signature Prep kit into our routine workflow for complex kinship analysis, an in-house naming system for both STR and microhaplotype SNP alleles was developed. This system makes use of a catalogue of alleles seen at each locus (provided as Supplementary Tables S1 and S2), allowing STR and SNP genotypes to be assigned based on the full amplicon sequences observed at each genetic marker. Using a simple VLOOKUP function in Microsoft Excel, it is possible to link a particular allele string sequence with the corresponding allele name.

The STR allele nomenclature uses the format vX_XX_SX, whereby “vX” denotes the flanking region variant, “XX” denotes the allele length, and “SX” denotes the ISFG minimum range sequence variant (which largely relates to the variation observed within the traditional “repeat-region”—see Figure 1 for more details). The separation of the prefix and the suffix with an underscore means that the length-based or minimum-range-based allele name can be easily extracted. This can be particularly useful for concordance checks with CE data or between different MPS-based STR assays, as well as data manipulation for research purposes (e.g., if comparing the power of length-based, repeat-region, and flanking-region allele differentiation). Due to the complex repeat structures of many STR loci, the “SX” suffix generally does not relate directly to any particular repeat pattern across alleles. However, when the same SNP change was seen in the flanking region of alleles of different lengths, these have been labelled with the same “vX”. This approach was chosen as mutations were often observed at the same position in alleles of differing lengths. Therefore, naming the flanking region variants in a way that corresponds to the specific SNP that was observed is logical.

The naming format applied to the SNP alleles was “X_XX_vX”, where “X” represents the nucleotide observed at the original bi-alleic SNP of interest, “XX” represents the nucleobases identified as “Detected Bases” by UAS, and “vX” represents the different sequence variants (see Figure 2 for details; all names given in Supplementary Table S2 correspond to the forward orientation of the sequence). As the number of bases reported by UAS varies across markers, whilst some loci may have alleles labelled “X_XX_vX”, others may have alleles labelled “X_XXXX_vX”. Currently, SNP and microhaplotype genotypes exported from UAS v1.3 are reported in either the forward or reverse strand orientation. In the presented nomenclature, all allele names have been converted into the forward strand orientation to increase cross-laboratory compatibly (both orientations are included in Supplementary Table S2). As with the STRs, this nesting naming system allows for specific designators, such as the bi-allelic allele name, to be easily extracted.

The use of SNP and/or microhaplotype loci is certainly not limited to the markers included in the ForenSeq DNA Signature Prep kit, with a number of studies independently demonstrating the utility of microhaplotypes for kinship analysis [42,43,44,45,46]. Therefore, a nomenclature system based on the UAS “Detected Bases” output is unlikely to be universal across all laboratories. However, the ability to isolate the bi-allelic information will once again facilitate participation in international proficiency testing exercises and collaborative projects. At present, the DNA Signature Prep kit is the only commercially designed solution that allows for the typing of microhaplotype loci. The benefit of a manufacturer-provided kit is that amplicon start and stop points will be standardised for all those using it. Therefore, it could also be supposed that laboratories wishing to implement this workflow would benefit from incorporating the UAS output directly into the naming system, i.e., the middle part of this example nomenclature. The use of version numbers (_vX) to symbolise sequence variation observed outside of the UAS “Detected Bases”, whilst beneficial when used within a single laboratory to increase the discriminating power of loci, would only be suitable for inter-laboratory use if there were to be a centralised database that was routinely updated with new variants. The value of such a resource is something that is likely to be discussed by the MWG going forward.

### 2.2. Accounting for Mutation Events in Kinship Pedigrees

**Consideration 2: When implementing a more complex allele naming system, accounting for mutations within likelihood ratio calculations may need to be applied independently of specialised kinship software solutions. Allele nomenclature that allows for the easy identification of the biological plausibility of a particular mutation event in terms of number of steps will be beneficial to the investigators.**

**Figure 3:**
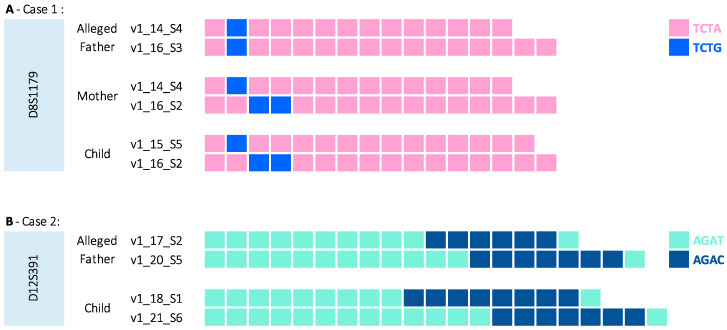
Sequence-based STR data for genetic inconsistencies in two paternity cases. Exclusion at D8S1179 in Case 1. The mother and the alleged father share the same length alleles at this locus, meaning that based on size alone it is impossible to tell whether the exclusion is maternal or paternal. Sequence information reveals that the allele 16 in the mother matches that of the child and differs from the allele 16 of the alleged father. Exclusion at D12S391 in Case 2. Based on length data, either of the alleged father’s alleles could have mutated to the child’s alleles. Sequence data reveals that only the alleged father’s 20 allele could have mutated to the child’s 21 allele, as the alleged father’s 17 allele could not have mutated to the child’s 18 allele.

The presence of genetic inconsistencies is most often of relevance to paternity or maternity investigations. Due to the way in which DNA is inherited by children from their parents, it is known that at any given locus, a parent and child should share one allele that is identical by descent (IBD). Therefore, if this is not the case, this can be referred to as a genetic inconsistency, which may point towards non-paternity, non-maternity or may indicate a possible mutation event. In these instances, MPS can once again provide an advantage over CE analysis. For example, there may be cases where from the CE data alone it is not possible to identify whether the mutation was maternal or paternal in nature. Figure 3A shows such a case, where at STR locus D8S1179 the CE genotypes obtained for the investigated paternity trio were Alleged Father: 14,16; Mother: 14,16; and Child: 15,16. If these are in fact the true parents of the child, then a mutation event must have occurred. This finding can be most problematic in cases where multiple exclusions between parents and children have been observed. From the MPS data obtained, however, it is possible to see the sequence structure of the alleles in question, which in turn can reveal that the child’s 16 allele has been inherited from the mother (v1_16_S2) rather than the father (v1_16_S3). Therefore, in order for the alleged father to be the true father, then a mutation event must have occurred. The plausibility of this is further supported by the sequence data, which shows that the alleged father’s 14 allele could theoretically mutate to the child’s 15 with only a single-step change. In other instances, MPS data might reveal whether a possible mutation event is biologically improbable (Figure 3B). A second case involves a paternity duo. At the locus D12S391, the CE genotypes were Alleged Father: 17,20; and Child: 18,21. Based on length data, either of the alleged father’s alleles could have mutated to produce the child’s genotypes. However, from the sequence data, it is possible to see that only a mutation of the v1_20_S5 to the v1_21_S6 allele is conceivable (Figure 3B). This mutation would result from the addition of one AGAT unit. Whereas, whilst v1_17_S2 and v1_18_S1 differ in length by one repeat unit, based on their structures, the 17 allele would need to lose one AGAT unit and simultaneously gain two AGAC units in order to become the 18 allele. Therefore, it is virtually impossible for these multiple mutation events to occur simultaneously.

When considering STR alleles using length-based differentiation, most often, a step-wise mutation model is used, which is relatively simple, as kinship software can numerically identify that “11” is one unit different from “12”. When including the sequence information, however, this process is much more complicated. Not only are allele names no longer strictly numerical, but the specific sequence structure of the alleles will also define which alternate alleles could result from a mutation event, as demonstrated in Figure 3B. Currently, it is unlikely that existing software solutions will be able to determine whether the mutation is plausible and subsequently apply a mutation rate based on the sequence-based allele name designations alone.

One potential work-around for implementing a step-wise mutation model in kinship calculations is to first conduct a manual evaluation of whether a mutation event could have occurred between two alleles. In these instances, a bracketed naming system will be advantageous. If the mutation is possible, these alleles could be renamed using their corresponding length-based designations in both the individuals’ genotypes and frequency file. This may allow for the mutation model to be applied automatically and for the mutation rate to be considered in the LR calculation. Alternatively, a mutation rate could be applied manually for that locus outside of the kinship software. Therefore, whilst MPS can provide useful information that has the potential to identify the mutational pathway when a genetic inconsistency is observed, further investigation into the most appropriate way to account for mutations within data analysis workflows is required.

### 2.3. Population Allele Frequency Databases

**Consideration 3: Laboratories will require suitable population allele frequency databases that are compatible with the chosen allele typing workflow. Due to the high proportion of rare alleles when utilising sequence data, there is a need for statistical adjustments to ensure that inaccurate frequency estimates do not generate misleading likelihood ratios in kinship investigations.**

Understanding how common an allele is within a population group of interest is a cornerstone of all forensic statistical analyses and evidence interpretation. The use of blood groups and protein polymorphisms for criminal and paternity investigations prompted the first widespread population studies to be conducted [47,48,49,50]. This was closely followed by studies of VNTR loci when DNA fingerprinting was first introduced [51]. At the time, Ranajit Chakraborty discussed the ideal specifications for VNTR population databases in his 1992 article, “Sample Size Requirements for Addressing the Population Genetic Issues of Forensic Use of DNA Typing”, which is arguably still relevant when applied to modern STR typing [52]. In this paper, Chakraborty emphasised that due to the hypervariable nature of these genetic markers, a great number of rare alleles are going to exist. Furthermore, the total number of alleles observed will increase as a function of the sample size, as will the number of rare alleles seen.

**Figure 4:**
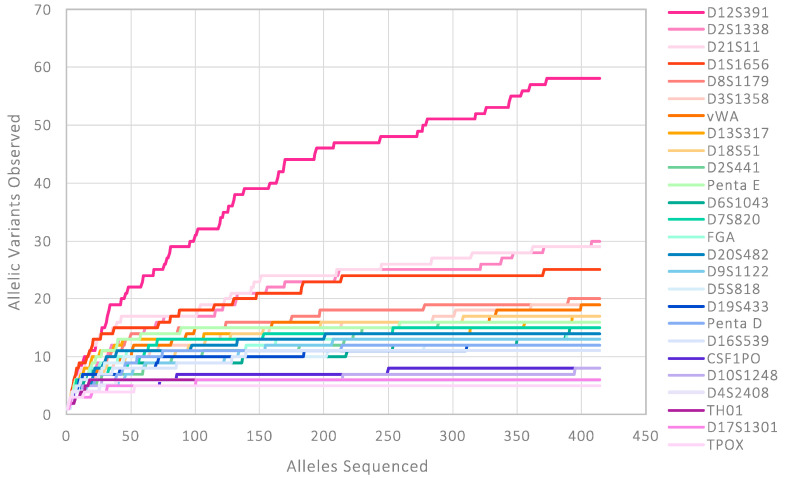
Number of distinct allelic variants observed in the White British population, when using the full amplicon sequence, against the total number of alleles sequenced for 26 STR loci targeted by the ForenSeq DNA Signature Prep kit. STR loci ordered by total number of allelic variants observed.

Sequenced-based STR data collected from the large-scale population study conducted at King’s College London (KCL) [17,18] supports this hypothesis. Figure 4 presents data generated from >200 White British individuals, showing that as the sample size increases, so does the number of allelic variants that are observed. Whilst for some of the less polymorphic STR loci the number of new variants observed begins to plateau, some loci are continuously revealing new variants at a notable rate. D12S391 is a well-documented example of this, with 58 sequence variants identified in this White British population data. A collaborative project between KCL and the National Institute of Standards and Technology (NIST) identified a total of 73 allelic variants when combining data from 1119 US Caucasian/White British individuals [53]. The contrast between the large number of sequence-based alleles observed at D12S391 and the 17 alleles obtained with length-based CE analysis presents new challenges for allele frequency databases.

**Figure 5:**
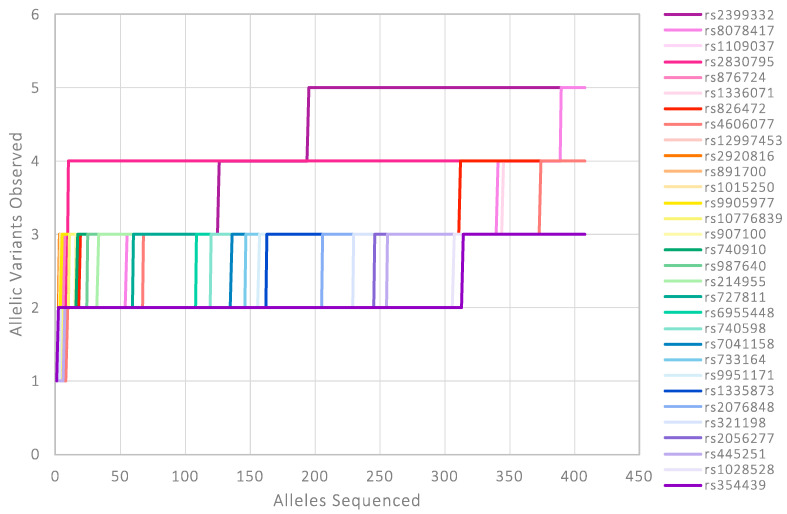
Number of distinct allelic variants observed in the White British population, when using the full amplicon sequence, against the total number of alleles sequenced for 31 SNP loci targeted by the ForenSeq DNA Signature Prep kit. Data only shown for the SNP loci that exhibited sequence variation in addition to the original SNP of interest. SNP loci ordered by total number of allelic variants observed.

Interestingly, this general pattern can also be somewhat seen for the identity-informative SNP loci (Figure 5). Targeted loci were originally chosen based on available bi-allelic frequency data. In a perfect scenario, the data for the selected loci would have displayed good levels of heterozygosity across global populations (i.e., the two alleles would ideally be equifrequent in most relevant population groups). However, as shown in the UK-relevant dataset, additional sequence variation within sequenced amplicons can be observed [19]. When plotting the number of distinct allelic variants observed vs. the number of alleles typed for the White British population, it is possible to see that, as more samples are processed, there is a rise in the number of allelic variants found in 31 of the 94 SNP loci. 

Chakraborty stated that if a frequency dataset aims to identify all possible alleles at a polymorphic locus, the number of individuals that would need to be tested from a single homogenous population would be impractically large. He consequently concluded that the main aim of a database should be to capture and accurately predict the frequency of the most common alleles and suggested that typing 100–150 individuals would be sufficient to achieve this. Additionally, he mathematically demonstrated that 300 individuals would be required to capture all variants seen at a frequency of >1% with at least 95% confidence [52]. John Butler later summarised that most published population studies typed between 150 and 200 individuals per population [54]. Various sets of regulations and recommendations for publishing population data have been proposed by FSI: Genetics and the DNA Commission of the ISFG [55,56,57]. At present, the recommended sample size for publication of STR population data generated with MPS is only 50 individuals, which is much smaller than the requirement of at least 500 individuals for CE data [57]. This number was chosen due to the novelty of MPS technology and to encourage the generation of sequence-level population data [58]. Furthermore, due to the higher expense of MPS analysis, large-scale population investigations can be very costly. Prior to publication, all STR data must also comply with the quality thresholds set out by STRidER, an online allele frequency database for autosomal STRs and quality control platform [57].

Due to the increased allelic diversity observed when differentiating STR alleles based on sequence, in comparison to length-based analysis, it could be hypothesised that MPS population studies containing a limited number of typed samples may be unsatisfactory and ineffective when used as a representative frequency database.

In his article, Chakraborty classed any allele observed at a frequency < 1% as rare. Subsequent publications went on to suggest the use of a minimum allele frequency (MAF), an idea that was further supported in a report by the National Research Council on Forensic DNA evidence [59,60]. A minimum allele frequency works on the principle that the confidence interval for a frequency calculated from a single observation within a reasonably large dataset is much greater than that of an allele that is observed approximately 20% of the time. Furthermore, an incorrect frequency estimate at a low level will have a far greater impact on a likelihood ratio calculation, both in a direct identification and kinship context. To demonstrate this point, if we consider a pair of individuals who believe they are half-siblings, at a locus of interest, they share a single allele that has a population frequency of 20.00% - the LR (for half-siblings vs. unrelated) at this locus would be calculated as the data being 1.13 times in favour of half-siblings. If the frequency of that allele is actually closer to 20.75%, the LR would be calculated as 1.10, so, overall, this frequency difference does not greatly impact this calculation. However, if the frequency of the shared allele is estimated as 0.25%, the LR for the locus would be 50.5 times in favour of half-siblings; but, if the true frequency of the allele was actually closer to 1%, this LR value would be reduced by nearly four times to 13. Therefore, unreliable estimation of these rare frequencies can greatly impact the LR values obtained. This highlights the difficult balance between maximising an LR in order to have the best chance of resolving a kinship case by utilising a method that allows for the detection of rare alleles but also making sure that the value of a shared rare allele is not overinflated.

Rather than using a fixed MAF value, it has been suggested that an allele needs to be observed at least five times within a dataset in order for the value to be a reliable estimate of the frequency, so, often, 5/2N (where N = number of samples in the dataset) can be used [54]. This means that the minimum allele frequency would continue to decrease as the number of samples typed for the frequency database increased. When using a MAF, there is a strong risk of overestimating the true frequency of rare alleles, which would particularly be the case for any observed private mutations, i.e., alleles that would only be found within a single pedigree. It would be very unlikely for these variants to be observed more than once in a database of unrelated individuals. However, the alternative is the risk of underestimating the allele frequency and overinflating LR values that may show inaccurately strong support for a relationship hypothesis (or potential match probability in a criminal identification scenario).

**Figure 6:**
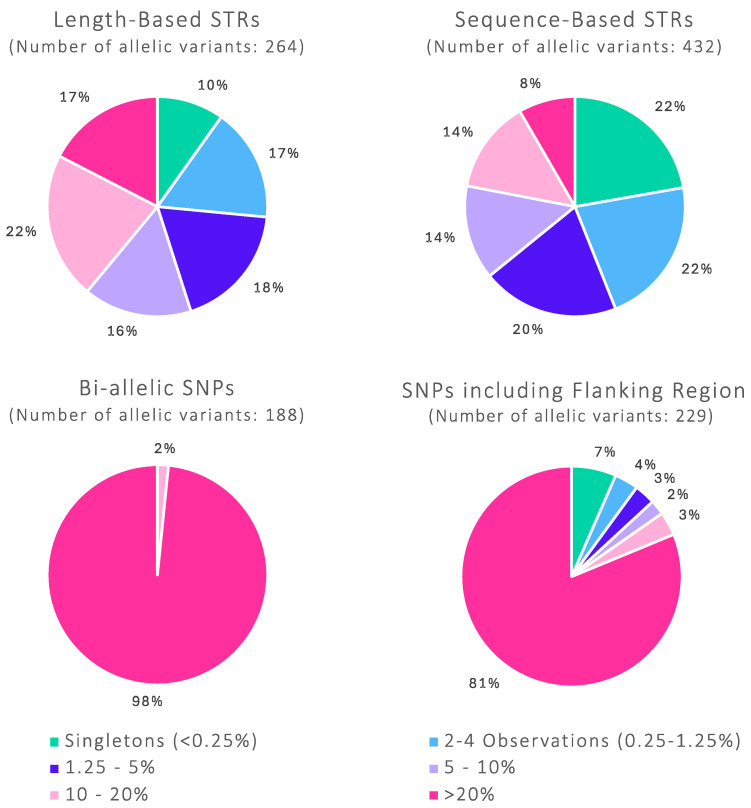
Relative frequency of alleles for the STR and SNP loci targeted by the ForenSeq DNA Signature Prep kit. Charts show the proportion of alleles with the frequency indicated in the legend: <0.25%, 0.25–1.25%, 1.25–5%, 5–10%, 10–20%, and >20% (where 20% = frequency of 0.2). Charts on the left-hand side show allele frequencies for STR and SNP using length-based and bi-allelic data. Charts on the right-hand side show allele frequencies for STR and SNP using sequence-based data, including flanking region variation. When sequence data is used, the proportion of “rare” alleles increases.

Figure 6 shows the relative frequency of STR and SNP alleles within the White British population for both length-based/bi-allelic data and full amplicon sequence-based data, demonstrating the impact of sequencing variation on the allele frequency distribution. When considering STR alleles by length only, 10% of variants (across all tested STR markers) were seen as a single observation. A further 17% had two to four observations in the dataset, meaning that just over a quarter of the total alleles observed are below a supposed minimum allele frequency (MAF) threshold of 5/2N (MAF = 0.0125, where N = 200). Differentiating alleles by sequence noticeably increases this proportion to 44%, with an equal proportion of 22% of alleles being singletons and the other 22% having two to four observations.

Current sequence-based STR frequency databases are unlikely to have captured all sequence variation, especially when considering population substructure and the randomness of sampling. Furthermore, as discussed above, the frequency of many sequence-based alleles that are contained within such a database are at risk of inaccurate estimation due to the range of allelic diversity. When coming across a new allele seen in a case, using a minimum allele frequency of 5/2N to evaluate the evidence is a feasible solution. However, trying to cap the minimum allele frequency of all rare alleles in the dataset is unlikely to be a practical approach. If there are a small number of rare alleles, a MAF cap is a reasonable and conservative approach that will likely have minimal impact on the frequency of the other alleles. However, as the presented STR sequence-based dataset shows, over 40% of all alleles are below the MAF threshold; therefore, adjusting all of these to 5/2N has the potential to greatly affect the frequency of all other alleles.

The distribution of allele frequencies for the SNP loci is unsurprisingly very different to that of the more polymorphic STRs when looking at the bi-allelic data. Only 2% of the bi-allelic SNP alleles have a frequency below 0.2, which corresponds to the least heterozygous loci (i.e., maximum heterozygosity would have both alleles at a frequency of 0.5). When including SNP flanking region sequence information, however, the new variants make up a small proportion overall, with 19% of the alleles having a frequency of <0.2. Of these sequence-based alleles, 7% are singletons, and a further 4% of alleles were observed two to four times within the dataset. Interestingly, of the 15 singletons within the White British population, 7 alleles have been seen more frequently in other tested population groups (Chinese, South Asian, North-East African, and West African), likely suggesting a common origin for these alleles [19]. Based on the rate and total number of new alleles detected at some of the SNP loci as well as the proportion of singletons, it could be supposed that at least some of the new variants are quite likely to be recent pedigree mutations and therefore will not be seen again in a larger population sample size of unrelated individuals. Overall, whilst observed at a far smaller proportion than the STRs, some of the sequence-based SNP alleles are still at risk of having inaccurate frequency estimates.

Due to the smaller proportion of rare alleles in the SNP dataset, a MAF-based frequency database method could be utilised in order to ensure that LR results are calculated in a conservative manner. However, a more universal method for dealing with rare alleles at all marker types may be a more reasonable and practical solution.

Therefore, for both STR and SNP data, in addition to cases with novel alleles, it may also be appropriate to implement a MAF cap in scenarios where an allele that is rare within the current database is shared by the tested individuals. Generally speaking, when interpreting results for any given kinship investigation, the reporting scientist should examine the individual LR values for all of the tested loci, and if the combined LR is heavily influenced by a single locus, processes should be in place to account for this. One such approach may be to manually apply a 5/2N frequency for the relevant allele and evaluate the impact on the combined LR in comparison to using the database frequency when reporting the case.

Periodically updating allele frequencies will enable population databases to be more representative of allelic diversity. It will be important that sample genotypes added to the population allele databases are chosen in an unbiased manner (i.e., not selectively prioritising profiles with rare alleles). As the number of alleles typed increases, the proportion of the allele dataset that can be classed as rare is likely to remain constant, but the absolute number of rare alleles is likely to increase. For rare alleles already observed, increasing N will allow for some these alleles to be observed more often and give more confidence to the calculated allele frequency. However, at the same time, it is likely that more, even rarer alleles will also be discovered. Generally speaking, whilst it may not be possible to predict when or how often these new variants occur, overall, it is of importance that we are able to estimate their frequency as accurately as is practical. Undoubtedly, a larger population database is likely to prove more useful both in terms of providing a more comprehensive picture of the variation present within the population but also in order to generate realistic but conservative frequencies for newly observed alleles.

This highlights the importance of developing strategies for dealing with rare alleles during kinship analysis. Laboratories will also need to independently validate their own procedures for building and maintaining appropriate allele frequency databases. When implementing MPS data into routine kinship investigations, laboratories should be aware of the limitations and unsuitability of applying a minimum allele frequency of 5/2N within a database when such a high proportion of alleles falls within this very low frequency range, as seen with the sequence-based STRs. Evaluation of the most appropriate approach for dealing with rarer alleles within frequency databases is an interesting topic for further exploration within the forensic community, especially with the advent of MPS. Additionally, it would be valuable to examine how often using the calculated frequencies for alleles with fewer than five observations is likely to result in misleading LR values that may impact the outcome of a case in kinship scenarios. In the meantime, it can be concluded that frequency estimates for rare alleles will have high uncertainty; as such, when observed in a case, manually applying a MAF to that allele is a suitably conservative and practical approach.

### 2.4. Combining Data from STR and SNP Loci and the Implementation of Genetic Linkage Corrections

**Consideration 4: It is necessary for statistical adjustments to be implemented into the calculation workflows to account for genetic linkage between loci when utilising expanded marker sets in kinship investigations.**

In kinship investigations involving more distant or complex relationship scenarios, there is often a need for the analysis of a greater number of genetic markers in order to achieve sufficient statistical support to resolve the case [9,61,62,63]. Being able to target both STR and SNP markers in one assay is a particular asset of MPS analysis; the added benefits of SNP loci, such as smaller amplicons and a low mutation rate, make them a valuable supplementary marker to STRs despite their lower individual polymorphism. Furthermore, analysing data from a larger number of loci that have fewer alleles has the potential to be less complex, allowing for more genetic information to more easily be obtained. With regard to the ForenSeq DNA Signature Prep kit, the sheer number of additional SNP loci that can be simultaneously analysed alongside STRs is the main factor enabling an increased resolving power to be achieved in kinship investigations. However, as the number of markers targeted increases, so does the chance of the loci being located close together on a chromosome, and hence the potential for genetic linkage between them. Genetic linkage refers to the propensity of two loci that are close together (i.e., molecularly close on the same chromosome) to be inherited together without recombination occurring between them. This can be described using centiMorgans (cM), a unit of genetic distance, whereby 1 cM equates to a 1% chance of a cross-over event occurring between two loci [64]. Two loci are said to be linked if they have a recombination rate (ϴ) of less than 0.5, meaning that the inheritance of alleles at the two loci can no longer be assumed to be independent.

It is known that accounting for genetic linkage in such circumstances will influence the LRs obtained during kinship investigations based on the fact that the underlying calculation process will be different compared to a method that treats markers as independent. Theoretically, it is possible for LR values in cases involving truly related pairs to both increase and decrease when accounting for linkage. This relies on the understanding that truly related individuals share alleles by descent and that the closer the genetic relationship between them, the more alleles a pair of individuals are likely to share. Assuming independence of loci, the statistical power of shared alleles at any locus will be multiplied together with all other loci using the product rule to generate a combined LR. When assuming linkage, however, if two individuals share alleles at multiple closely linked loci, accounting for genetic linkage in this scenario will strengthen the overall combined LR.

When DNA is inherited, recombination during meiosis generates different combinations of DNA segments on each chromosome pair that will be shared by closely related individuals. Generally speaking, the more distantly related a pair, the shorter the length of the shared segments. Evidence of shared alleles across many consecutively linked loci therefore implies that that segment of DNA has been inherited as one from a shared origin, supporting the hypothesis that those alleles are identical by descent (IBD). However, if accounting for linkage actually highlights that the individuals share an allele at one locus, but they do not share any alleles at the linked loci, this in turn will reduce the support for the shared alleles being IBD, instead indicating that the alleles are identical by state (IBS) by chance alone, which may reduce the overall LR.

Consequently, for kinship investigations, it is important to understand whether the impact of accounting for linkage alters LR values significantly enough to necessitate the modification of data analysis workflows from those currently employed in kinship laboratories that are using data from linked loci. Various studies have shown that accounting for genetic linkage on an individual case basis can make a notable difference in the final LR value obtained [5,61,62,65,66]. This in turn confirms the need for alternative statistical analysis methods that can incorporate genetic linkage corrections. Whilst initially some laboratories dealt with linked loci by excluding one of the linked markers from the calculations [67,68,69], this is not a suitable or feasible option when the purpose of targeting a larger number of markers is to provide more power when evaluating distant relationship scenarios. Therefore, to obtain the most accurate LR values, genetic linkage between linked loci should be accounted for by considering the chance that a recombination event will take place between them.

One of the early studies that focused on genetic linkage was conducted by Nothnagel et al. in 2010 [61]. This study investigated the impact of linkage corrections in kinship calculations when analysing 34 STRs. It was recommended that on an individual case basis, the exact LR calculation incorporating recombination rates should be carried out. These conclusions have been mirrored in the small number of studies that have since been conducted using expanded marker sets with MPS [62,65].

Historically, accounting for genetic linkage in kinship calculations has been viewed as highly problematic and complex. This was in part due to the lack of available user-friendly software and adequate data resources in contrast to those that had been well-established for kinship calculations using unlinked markers.

The study by Nothnagel et al. highlighted the need for more accurate estimates of the genetic distances between loci in order to improve the accuracy of the LR calculations and went on to propose the generation of a genetic map of forensically relevant STRs. The necessity of this additional resource presented itself as one of the main complications for incorporating genetic linkage corrections within kinships calculations.

A study by Phillips et al. in 2011 went on to produce such a genetic map for forensic STRs using Kosambi-adjusted genetic distances based on SNP proxies from high-density SNP data [64]. Subsequently, a number of software packages and computing tools have been designed to carry out linkage analysis, which in turn require information about genetic distances between the tested loci as a data input file. Therefore, the work of Phillips et al. has complemented the development of such data analysis pipelines.

One such program, FamLink2, has a default genetic map included in the software download package that contains genetic distance information for a large number of forensically relevant loci. Therefore, theoretically, for most laboratories wishing to implement expanded marker sets that already use Familias, no additional work would be required other than to validate the use of FamLink2 as an extension to their workflow.

It is also possible to use a custom genetic map containing map locations for the specific loci analysed by a laboratory via the “Quick analysis” option in FamLink2. A genetic map file that contains both the STR and identity SNP markers targeted by the DNA Signature Prep kit and other commonly used forensic STRs typed by commercial CE kits is included in Supplementary Table S3. This file was generated using a fine-scale combined physical-linkage map produced by researchers at Rutgers University [70]. This genetic map can be easily expanded to suit the requirements of a laboratory by extracting the linkage-map locations from the Rutgers dataset with the appropriate rs number.

## 3. Conclusions

Whilst massively parallel sequencing technology has the potential to be highly beneficial when applied to kinship testing, there are aspects of this alternative workflow that require consideration before it can be incorporated into routine casework.

Some of these factors may not be immediately apparent to laboratories currently working with CE-based systems; however, the empirical data and casework experience presented here highlight the need for considerable, although not necessarily challenging, procedural adjustments when using this more advanced technology. This article outlines four key areas of interest for analysts wanting to incorporate MPS into their kinship workflows and provides associated resources and data to aid laboratories in this implementation and validation process.

## Data Availability

The original contributions presented in this study are included in the article/Supplementary Materials. Further inquiries can be directed to the corresponding author.

## Supplementary Materials

The following supporting information can be downloaded at https://www.mdpi.com/article/10.3390/genes16030238/s1, Table S1: Catalogue of STR sequences; Table S2: Catalogue of SNP sequences; Table S3: Custom genetic map.
